# Developmental fluoxetine exposure affects adolescent and adult bone depending on the dose and period of exposure in mice

**DOI:** 10.14814/phy2.15881

**Published:** 2023-11-29

**Authors:** Hannah P. Fricke, Chandler J. Krajco, Molly J. Perry, Katelyn M. Desorcy‐Scherer, Lella A. Wake, Julia F. Charles, Laura L. Hernandez

**Affiliations:** ^1^ Endocrinology and Reproductive Physiology Program University of Wisconsin‐Madison Madison Wisconsin USA; ^2^ Department of Animal and Dairy Sciences University of Wisconsin‐Madison Madison Wisconsin USA; ^3^ School of Nursing University of Wisconsin‐Madison Madison Wisconsin USA; ^4^ Departments of Orthopedics and Medicine Brigham and Women's Hospital Boston Massachusetts USA

**Keywords:** body composition, fluoxetine, lactation, offspring

## Abstract

At the end of gestation, fetal skeleton rapidly accumulates calcium, and bone development continues in offspring postnatally. To accommodate, maternal skeletal physiology is modulated in a serotonin‐dependent manner. Selective serotonin reuptake inhibitors (SSRIs) are generally considered safe for treatment of major depressive disorder, postpartum depression, and other psychiatric illnesses during the peripartum period, but because serotonin affects bone remodeling, SSRIs are associated with decreased bone mass across all ages and sexes, and the impact of SSRIs during fetal and postnatal development has not been fully investigated. In the present study, our aim was to examine developmental fluoxetine exposure on offspring skeleton and to assess varying degrees of impact depending on dose and window of exposure in short‐term and long‐term contexts. We established that a low dose of lactational fluoxetine exposure caused a greater degree of insult to offspring bone than either a low dose during fetal and postpartum development or a high dose during lactation only in mice. We further discovered lasting impacts of developmental fluoxetine exposure, especially during lactation only, on adult bone and body composition. Herein, we provide evidence fluoxetine exposure during early development may have detrimental effects on the skeleton of offspring at weaning and into adulthood.

## 1 INTRODUCTION

Postpartum depression (PPD), defined as a major depressive episode that occurs during pregnancy or the first 4 weeks post‐parturition, affects 10%–15% of people (American Psychiatric Association, 2013; Kroska & Stowe, 2020). There can be severe consequences of PPD on both parent and infant, including the disruption of bonding, issues with lactation initiation, and early cessation of lactation (Dennis & McQueen, 2009; Goodman & Gotlib, 1999). The World Health Organization recommends exclusively breastfeeding an infant for the first 6 months of life, highlighting the benefits to both members of the breastfeeding dyad (Infant and young child feeding [Online], 2021). Selective serotonin reuptake inhibitors (SSRIs) are frequently used as the first‐line treatment for depression and PPD both among the general population, and pregnant and lactating individuals (Cooper et al., 2007). All SSRIs are transported across the placenta at varying rates and are present in milk, resulting in in utero and lactational SSRI exposure; however, untreated depression or PPD in the peripartum period carries its own risks (di Scalea & Wisner, 2009; Ewing et al., 2015; Heikkinen et al., 2003; Hendrick et al., 2003; Yoshida et al., 1998). Depression during pregnancy has been associated with higher rates of miscarriage, preterm delivery, low birth weights, prolonged labor, and preeclampsia (Hermon et al., 2019). During the postpartum period, maternal depression is implicated in emotional, behavioral, and cognitive difficulties later in life for the exposed offspring (Biederman et al., 2001; Pilowsky et al., 2006). Therefore, doctors and their patients must weigh the risks of untreated depression or PPD against the risks of SSRI usage during the peripartum period.

Aside from paroxetine (Paxil™), which has been previously shown to be associated with congenital and cardiac malformations, there is conflicting evidence on whether SSRI exposure is linked with teratogenic effects, and it is difficult to distinguish between the effects of antidepressant exposure or the effects of the underlying psychiatric disorder itself (Bérard et al., 2016; Einarson et al., 2009; Wogelius et al., 2006). Further, there is evidence that SSRI usage during pregnancy is associated with infants that are small for gestational age or born preterm, once again with the caveat that the findings may have been confounded by the effects of maternal depression itself (Oberlander et al., 2006; Toh et al., 2009).

In 2018, fluoxetine (Prozac™) was the 20th most common medication prescribed in the United States and remains one of the most commonly prescribed antidepressants (Fuentes et al., 2018). Fluoxetine was the first SSRI introduced in the United States in 1987 and remains widely used among both the general population, as well as during pregnancy and lactation (Bandoli et al., 2020; Wong et al., 2005). The relative infant dose, or the dose received by the infant via breast milk in comparison with the maternal dose, is considered to be a cause for concern if it exceeds 10% (Hale & Rowe, 2016). The relative infant dose of fluoxetine and its active metabolite, norfluoxetine, varies across studies and by dose but generally remains below this 10% threshold (Heikkinen et al., 2003; Taddio et al., 1996; Yoshida et al., 1998). Still, there are concerns about the safety of fluoxetine exposure during the peripartum period and the short‐ and long‐term effects on the offspring.

There is a relationship between fetal and/or lactational developmental exposure to fluoxetine and the effect on bone development in the offspring. When the serotonin transporter, SERT, was inhibited in growing mice, it resulted in reduced bone mass and altered bone architecture (Warden et al., 2005). Further, we have previously shown that in utero and lactational exposure to 20 mg/kg fluoxetine resulted in a reduced femoral length and bone mineral density, as well as a negative impact on trabecular and cortical characteristics at weaning (Weaver et al., 2019). Taking these findings into consideration, we aimed to examine the effect of exposure to different doses of fluoxetine, the critical window in which exposure has the greatest impact and the long‐term implications of developmental fluoxetine exposure on the offspring.

## 2 MATERIALS AND METHODS

### 2.1 Experiments to examine the effect of timing and dose of fluoxetine on offspring bone at weaning

#### 2.1.1 Animals

All experiments were approved by the Research Animal Care and Use Committee at the University of Wisconsin‐Madison and were performed under protocol number A005789‐R01‐A03 and protocols were strictly followed. Female C57BL/6 mice were obtained from Jackson Laboratories at 5 weeks of age ± 3 days (stock #00064, Jackson Laboratories, Bar Harbor, ME). Mice were housed in groups in an environmentally controlled facility for biological research in the Biochemistry department vivarium at the University of Wisconsin‐Madison. Mice were maintained at a temperature of 25°C and a humidity of 50%–60% on a 12‐h light/dark cycle with food (Envigo‐Teklad #2018) and water access ad libitum.

Beginning at 6 weeks of age, female mice were mated overnight with a male C57BL/6 mouse also obtained from Jackson Laboratories. Pregnancy was determined by the presence of a vaginal plug, at which point the female mice were housed individually. On the first day of gestation (E0), mice were randomly assigned to receive sterile saline or either 2 mg/kg or 20 mg/kg fluoxetine hydrochloride (S6319; Sigma‐Aldrich, St. Louis, MO, USA) during lactation only or during gestation and lactation. reconstituted in sterile saline. Treatment was administered via intraperitoneal injection between the hours of 0800 and 0900 daily. The number and weight of the pups were recorded daily from P0 to the end of lactation (D21). The litters were not standardized due to the effect of fluoxetine on litter size and pup mortality (Domingues et al., 2022). Previously, we examined the effect of 20 mg/kg fluoxetine exposure during gestation and lactation at weaning and due to our previous findings and the negative pregnancy outcomes associated with 20 mg/kg administration during gestation, we chose not to treat any dams with 20 mg/kg fluoxetine during gestation (Domingues et al., 2022; Weaver et al., 2019).

**FIGURE 1:**
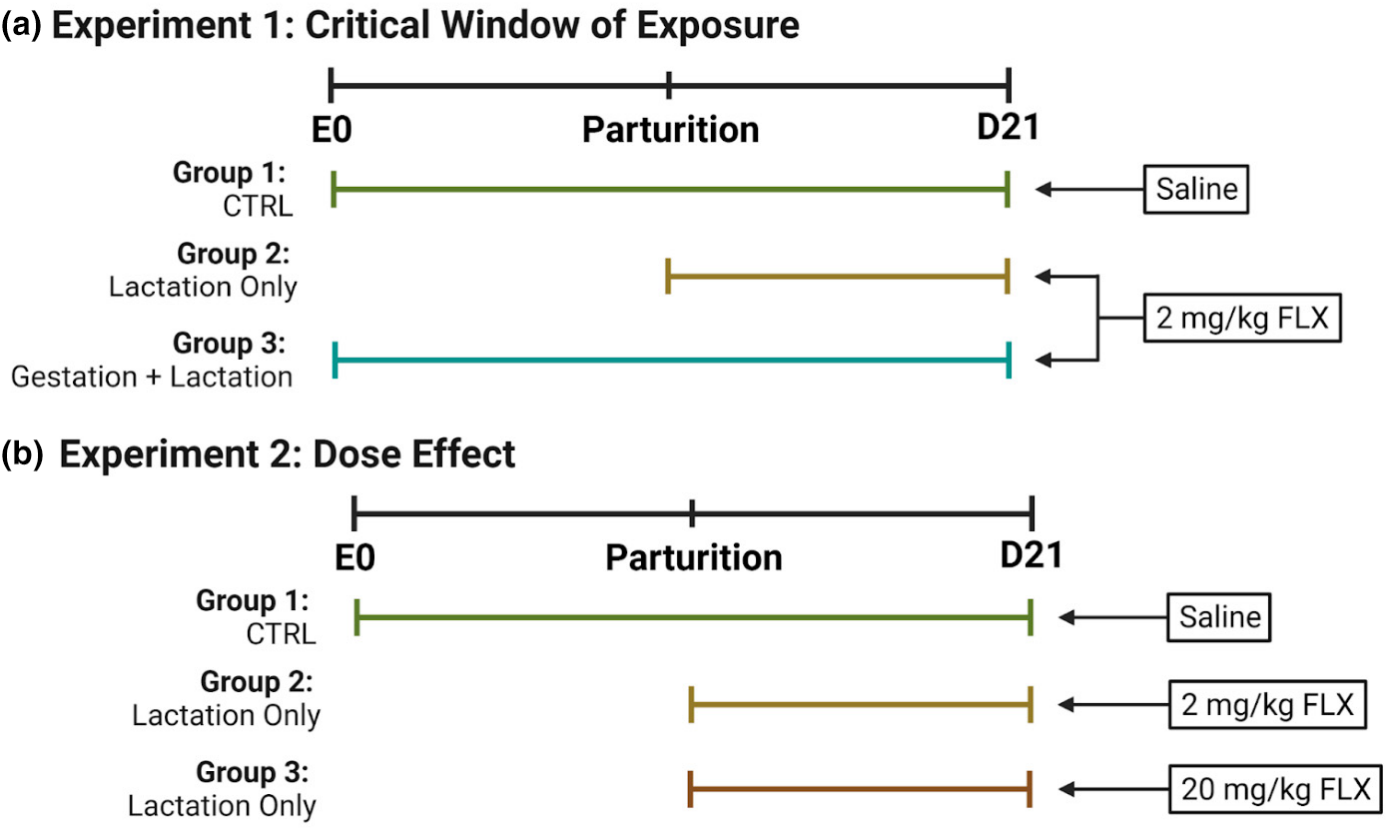
Dosing regimen of Experiments 1 and 2. (a) Offspring were exposed to saline during the entire peripartum period beginning with the first day of gestation (E0), to 2 mg/kg fluoxetine from parturition through the end of lactation (D21), or to 2 mg/kg fluoxetine during both gestation and lactation. (b) Offspring were exposed to saline during the entire peripartum period, to 2 mg/kg fluoxetine during lactation, or to 20 mg/kg fluoxetine during lactation.

**EXPERIMENT 1** (Figure 1): In order to determine the critical window of fluoxetine exposure during development, dams were administered 2 mg/kg fluoxetine either beginning on the first day of lactation (D1) until D21 (lactation only; *n* = 10) or beginning on P0 until D21 (gestation and lactation; *n* = 8). Control dams were treated with saline from P0 to D21 (*n* = 9).

**EXPERIMENT 2** (Figure 1): To further investigate the effect of dose on dams treated with fluoxetine during lactation, dams were administered 20 mg/kg fluoxetine beginning on D1 until D21 (*n* = 8). The control group and the 2 mg/kg lactation‐only group are the same groups described in Experiment 1.

#### 2.1.2 Sample collection

The number of pups and total weight of the litter were measured beginning on D1 through D21. At weaning (D21), 1–2 male and female mice were collected from each litter and euthanized via carbon dioxide followed by cervical dislocation. Blood was collected via cardiac puncture and was centrifuged at 3000 × *g* for 20 min to isolate serum. Serum samples were stored at −80°C until time of assay. The head and skin were removed, and the carcasses were stored in 70% ethanol until microCT analysis.

#### 2.1.3 MicroCT analysis

The femurs of the exposed dams were analyzed by micro‐computed tomography (microCT) using a Sanco Medical μCT 35 system with an isotropic voxel size of 7 μm. Scans were conducted in 70% ethanol using an X‐ray tube potential of 55 kVp, an X‐ray intensity of 0.145 mA, and an integration time of 400 ms. Digital calipers were used to measure femoral length. Cancellous bone analysis was measured via a selected region beginning 0.14 mm proximal to the growth plate and extending 1.4 mm proximally. Cortical parameters were calculated via a selected region centered at the midpoint of the femur and 0.6 mm in length. Cortical and trabecular bone were distinguished via a semi‐automated contouring approach. The region of interest was selected using a global threshold that set the bone/marrow cutoff at 512 mgHA/cm^3^ for trabecular bone and 871.8 mgHA/cm^3^ for cortical bone. The three‐dimensional microstructural properties of the bone, which include the bone volume fraction (BV/TV), trabecular thickness (Tb.Th), trabecular number (Tb.N.), trabecular separation (Tb.Sp.), midshaft bone volume fraction (M.BV/TV), connectivity density (Conn.D), tissue mineral density (TMD), bone mineral density (BMD), cortical area (Ct.Ar), cortical thickness (C.Th), and the periosteal perimeter (Ps.Pm) were calculated with software supplied by the manufacturer and were reported according to consensus guidelines on rodent microCT (Bouxsein et al., 2010).

#### 2.1.4 Fluoxetine assay

Serum fluoxetine and its active metabolite, norfluoxetine, were measured with a forensic fluoxetine ELISA kit (107,619; Neogen, Lexington, KY, USA) according to the manufacturer's instructions. Samples were diluted 1:10 to fit within the standard curve, which was prepared with fluoxetine hydrochloride (S6319; Sigma‐Aldrich, St. Louis, MO, USA). The cross‐reactivity for fluoxetine is 100% and 67% for norfluoxetine; data are presented as the combination of fluoxetine and norfluoxetine concentrations.

#### 2.1.5 Statistics

All statistics were conducted with GraphPad Prism 9 (Version 9.5.1). Analyses between the three treatment groups without the effect of time were performed using a one‐way ANOVA with Tukey's multiple comparison test to analyze the differences between treatment groups. When data were not normally distributed, a Kruskal–Wallis test was performed for nonparametric data with Dunn's multiple comparison test. Analyses with multiple time points were performed using a two‐way ANOVA with Tukey's multiple comparison test to analyze the differences between treatment groups. For analyses, differences among means were considered significant if *p* < 0.05 or a tendency if 0.05 < *p* < 0.1. All values are reported as mean ± SD and bars represent the mean.

### 2.2 Experiment to investigate the long‐term effects of fluoxetine exposure on offspring bone

#### 2.2.1 Animals

All experiments were approved by the Research Animal Care and Use Committee at the University of Wisconsin‐Madison and were performed under protocol number A005789‐R01‐A03. Mice were housed in the same facility as described in the previous experimental design.

Beginning at 6 weeks of age, female mice were mated overnight with a male C57BL/6 mouse also obtained from Jackson Laboratories. Pregnancy was determined by the presence of a vaginal plug, at which point the female mice were housed individually. On the first day of pregnancy (E0), mice were randomly assigned to receive sterile saline or 20 mg/kg fluoxetine hydrochloride (S6319; Sigma‐Aldrich, St. Louis, MO, USA) reconstituted in sterile saline daily during lactation only or during gestation and lactation. Treatment was administered via intraperitoneal injection between the hours of 0800 and 0900 daily. The number and weight of the pups were recorded daily from P0 to the end of lactation (D21). The litters were not standardized.

#### 2.2.2 Sample collection

The number of pups and total weight of the litter were measured beginning on D1 through D21. On D21, 1–3 male and female mice from each litter were weaned. At 12 weeks of age, the mice were euthanized via carbon dioxide followed by cervical dislocation. Blood was collected and stored using the same method as described above. The right femur of each animal was collected, and snap‐frozen in liquid nitrogen before being stored at −80°C until RNA was extracted. The head and skin were removed, and the carcasses were stored in 70% ethanol until microCT analysis.

#### 2.2.3 Femur RNA extraction and RT‐PCR

Total RNA was extracted from the femur using TRI‐Reagent (NC9330796; Molecular Research, Cincinnati, OH), and RNA was reverse transcribed (1 μg) to cDNA via the Applied Biosystems High Capacity cDNA Reverse Transcription Kit (4368814; Applied Biosystems, Foster City, CA). Quantitative RT‐PCR was performed using the CFX96 Touch Real‐Time PCR Detection System (Bio‐Rad Laboratories, Rodeo, CA), and reaction mixtures and cycling conditions were performed as previously described (Laporta et al., 2013). Primers were designed to span exon–exon junctions with an optimal annealing temperature of 60°C, and amplification efficiencies were accepted within a range of 95%–105% (Table 1). Primer specificity was determined by the presence of a single temperature dissociation peak.

**TABLE 1:** Primer sequences used for RT‐qPCR.

Gene	Forward primer 5′ → 3′	Reverse primer 3′ → 5′
*5ht2a*	AGCCAGCACAGACTTCAACC	GGAATCCCCTCTCTTTGAGC
*5ht2b*	CAATCATCCTCCTCGATACCC	GAAGCCATCAGATCTACTTTAGCC
*M‐csf*	CGAATGTTCTCCCACTTCCT	TGGACAATCAAAGGCTGAGG
*Mcp1*	CCAAAGAAGCTGTAGTTTTTG	GGTTCCGATCCAGGTTTTTA
*Mmp13*	CCGAACTTAACTTACAGGATTG	GGTGTCACTCAGACCAGACC
*Opg*	AAGCTGGAACCCCAGAGC	GTGCTGCACTTCGTGTGTTT
*Pdk4*	GGATGGAAGGAATCAAAGCA	CACTGGCTTTTTGAGTGCAA
*Pth1r*	GGACAGATGGACCAAGAAGC	TTGAGCACAACACAGGAAGC
*Rank*	CAGGACAGGGCTGATGAGAG	CCGCTAGAGATGAACGTGGA
*Rankl*	GGAGGATGAAACAAGCCTTTG	ACATCCAACCATGAGCCTTC
*Runx2*	ATGCTTCATTCGCCTCACAAA	GCACTCACTGACTCGGTTGG
*Sert*	ATCACGCTGGGTTTGGATAG	ATGACCACGATGAGCACAAA
*Tph1*	CCCGGAAATCAAAGCAAAG	CTTCCTTCGCAGTGAGCTG
*Trap*	CGACAAGAGGTTCCAGGAGA	TGCCAAGGTGATCATGGTTT

Abbreviations: *5ht2a*, 5‐hydroxytryptamine receptor 2A; *5ht2b*, 5‐hydroxytryptamine receptor 2B; *M‐csf*, colony stimulating factor 1; *Mcp1*, monocyte chemoattractant protein‐1; *Mmp13*, matrix metallopeptidase 13; *Opg*, osteoprotegrin; *Pdk4*, pyruvate dehydrogenase kinase 4; *Pth1r*, parathyroid hormone 1 receptor; *Rank*, receptor activator of nuclear factor κΒ; *Rankl*, receptor activator of nuclear factor κΒ ligand; *Runx2*, runt‐related transcription factor 2; *Sert*, serotonin transporter; *Tph1*, tryptophan hydroxylase 1; *Trap*, tartrate‐resistant acid phosphatase.

#### 2.2.4 DEXA analysis

Bone densitometry and body composition of the exposed dams were measured using dual‐energy X‐ray absorptiometry (DEXA) via a PIXImus2 Mouse Densitometer (GE Medical Systems, Madison, WI) as previously described (Lee et al., 2014). Quality control measurements were performed with a phantom before each session, and the mice were anesthetized during each measurement via isoflurane inhalation. Measurements were collected at 6 weeks of age as a baseline measurement, then at 9 and 12 weeks of age. Analysis of the scans was performed using Lunar Piximus Software using auto‐thresholding. Areal bone mineral density (BMD) of the femur and total body was measured, along with the total tissue mass (TTM) and % fat of the total body.

#### 2.2.5 MicroCT analysis

At 12 weeks of age, microCT analysis was performed as described above.

#### 2.2.6 Assays

Insulin‐like growth factor 1 (IGF‐1) concentrations were measured via R&D Systems quantikine ELISA kit (MG100; R&D Systems, Minneapolis, MN, USA) per manufacturer's instructions. Samples were diluted 1:500 to fit within the standard curve, and measurements with a CV value above 10 were excluded from analysis. Serum procollagen I intact N‐terminal (P1NP) concentrations were measured via Immunodiagnostics Systems enzyme immunoassay (AC‐33F1; Immunodiagnostics Systems, Tyne and Wear, United Kingdom) per manufacturer's instructions. Samples were diluted 1:10 to fit within the standard curve.

#### 2.2.7 Statistics

Statistical analysis for this experiment was performed as described previously.

## 3 RESULTS

### 3.1 Fluoxetine exposure during development does not impact pup growth prior to weaning

**FIGURE 2:**
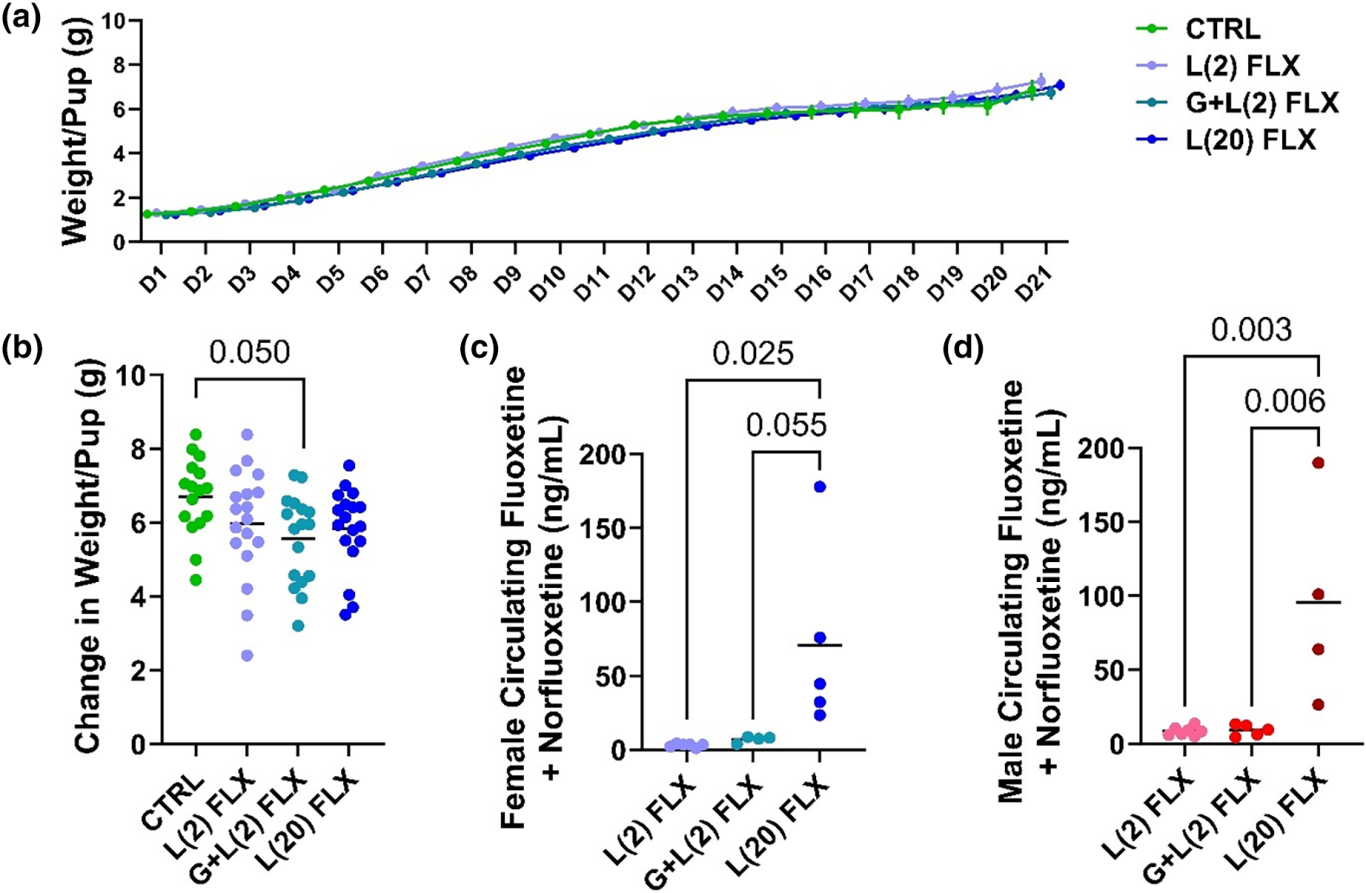
Average pup weight and circulating fluoxetine + norfluoxetine concentrations of offspring developmentally exposed to fluoxetine. C57BL/6J dams were administered sterile saline (*n* = 14), 2 mg/kg fluoxetine during lactation (*n* = 18), 2 mg/kg fluoxetine during gestation and lactation (*n* = 19), or 20 mg/kg fluoxetine during lactation (*n* = 18). The daily litter weight was recorded from birth to weaning, and the average pup weight was obtained by dividing the litter weight by the number of pups. (a) At weaning, the change in average pup weight relative to average pup weight at D1 was measured. (b) The circulating concentrations of fluoxetine and its active metabolite, norfluoxetine, were measured in the (c) female L(2) FLX (*n* = 6), G + L(2) FLX (*n* = 4), and L(20) FLX (*n* = 5) offspring and the (d) male L(2) FLX (*n* = 7), G + L(2) FLX (*n* = 5), and L(20) FLX (*n* = 4) offspring at weaning. Bars represent the mean; *p* < 0.05 is considered significant and 0.1 < *p* < 0.05 is considered a tendency.

**FIGURE 3:**
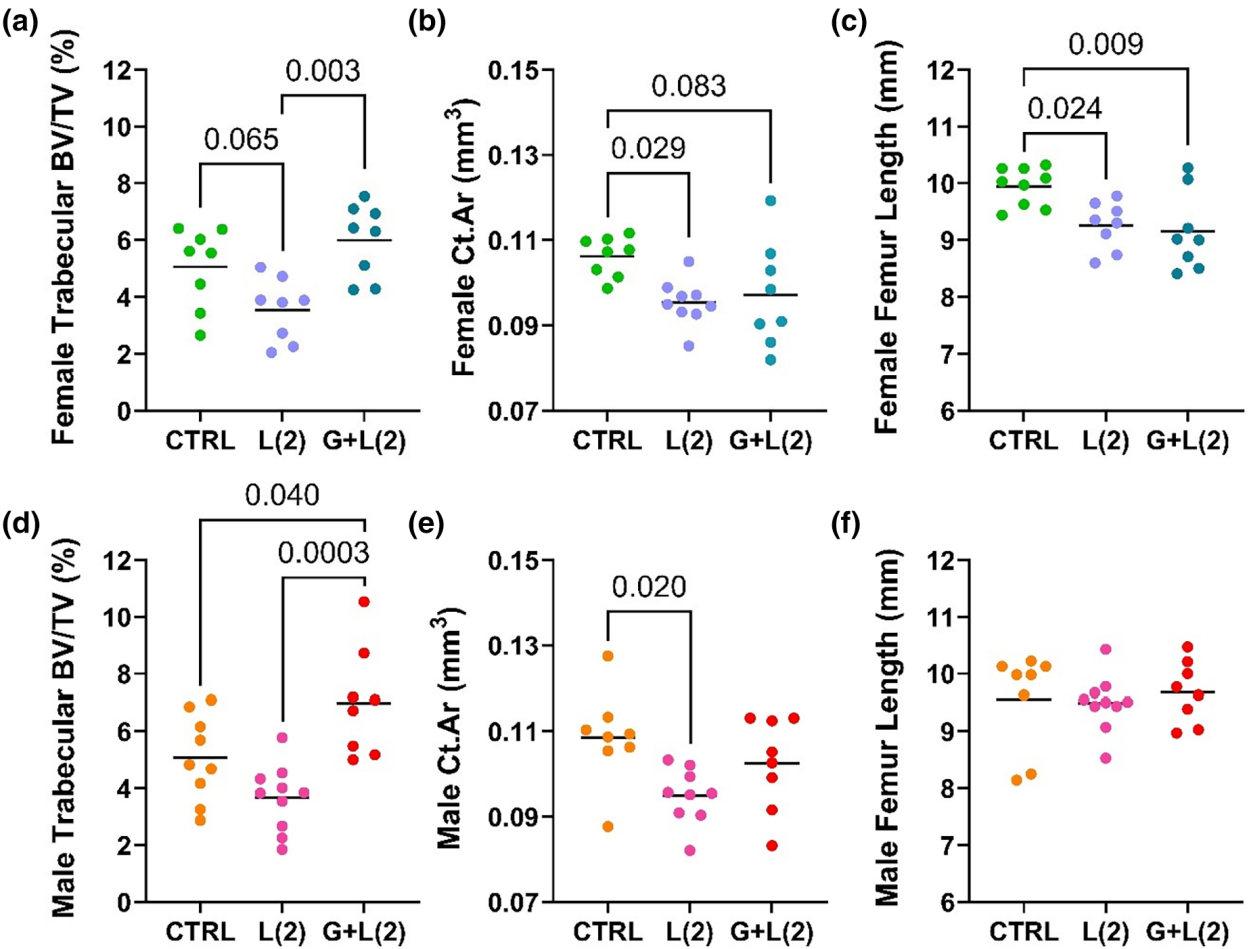
Fluoxetine exposure during lactation or during both gestation and lactation has differential and sex‐specific impacts on cortical and trabecular characteristics at weaning. C57BL/6J dams were administered 2 mg/kg fluoxetine during either lactation alone or both gestation and lactation. MicroCT was used to measure trabecular (control: *n* = 8 female, *n* = 9 male); L(2) FLX: *n* = 8 female, *n* = 10 male; G + L(2) FLX: *n* = 8 female, *n* = 8 male) and cortical (control: *n* = 8 female, *n* = 8 male; L(2) FLX: *n* = 9 female, *n* = 10 male, and G + L(2) FLX: *n* = 8 female; *n* = 8 male) parameters in the female and male offspring. The trabecular BV/TV (a,d), cortical area (b,e), and femur length (c,f) were measured. Bars represent the mean; *p* < 0.05 is considered significant and 0.1 < *p* < 0.05 is considered a tendency.

Firstly, the average weight per pup throughout lactation and at weaning was examined. The number of pups and the total litter size were measured daily, and the average weight per pup was determined by dividing the total litter weight by the number of pups (Figure 2a). There were no significant differences in average pup size at any point throughout lactation. In order to determine the average weight gain per pup throughout lactation compared to birth, the average weight at weaning was assessed relative to the average weight at birth (Figure 2b). The G + L(2) FLX group had significantly less average weight gain than the control pups (*p* < 0.05), but there were no significant differences between the other groups. To determine the degree of fluoxetine exposure in each group, the circulating concentrations of fluoxetine and norfluoxetine were measured in both the female and male mice at weaning. There was no detectable circulating fluoxetine + norfluoxetine in the control groups, and therefore, those data are not shown. In the females, there was significantly less circulating FLX + NFLX in the L(2) FLX mice compared with the L(20) FLX mice (*p* < 0.05) and a tendency to have lower FLX + NFLX in the G + L(2) FLX group compared with the L(20) FLX group (*p* < 0.1) (Figure 3c). In the male mice, both the L(2) FLX and G + L(2) FLX groups had significantly less circulating FLX + NFLX than the L(20) FLX group (*p* < 0.01 and *p* < 0.01, respectively) (Figure 3d).

### 3.2 Exposure to a low dose of fluoxetine during lactation alone or gestation and lactation impacted cortical and trabecular bone in a sex‐dependent manner at weaning

MicroCT analysis was used to examine the differential effects on cortical and trabecular parameters of 2 mg/kg fluoxetine during lactation or during both gestation and lactation. There were sex‐specific changes in both cortical and trabecular parameters between the different dosing periods. In the female offspring, there was a tendency for the L(2) FLX group to have a lower BV/TV (3.544% ± 1.105%) compared with the controls (5.064% ± 1.408%; *p* < 0.1), and to be significantly lower compared with the G + L(2) FLX group (5.994% ± 1.284%; *p* < 0.01) (Figure 3a). In the males, the BV/TV of the G + L(2) FLX group (6.989% ± 1.896%) was significantly greater compared with both the control group (5.067% ± 1.501%) and the L(2) FLX group (3.663% ± 1.164%; *p* < 0.05 and *p* < 0.001, respectively) (Figure 3d). In the female offspring, the cortical area was significantly lower in the L(2) FLX mice (0.095 ± 0.005 mm^2^; *p* < 0.05) and tended to be lower in the G + L(2) FLX mice (0.097 ± 0.012 mm^2^; *p* < 0.1) compared with the control females (0.106 ± 0.005 mm^2^) (Figure 3b). Similarly, in the male offspring, the cortical area was lower in the L(2) FLX mice (0.095 ± 0.007 mm^2^; *p* < 0.05) compared with the control mice (0.109 ± 0.011 mm^2^), but there were no differences between the G + L(2) FLX males (0.103 ± 0.011 mm^2^) and the control males, nor were there differences between the fluoxetine‐treated groups (Figure 3e). When comparing the femur length, both the L(2) FLX (9.256 ± 0.418 mm; *p* < 0.05) and the G + L(2) FLX groups (9.149 ± 0.687 mm; *p* < 0.01) had shorter femurs compared with the controls (9.948 ± 0.334 mm) in the female offspring (Figure 3c).However, there was no difference between the control (9.558 ± 0.860 mm), L(2) FLX (9.492 ± 0.484 mm), or G + L(2) FLX (9.684 ± 0.538 mm) groups in the male offspring (Figure 3f).

MicroCT analysis was used to further examine the cortical and trabecular parameters in both the female (Table 2) and male (Table 3) offspring at weaning. In the female mice, several cortical and trabecular parameters were significantly impacted in the L(2) FLX group when compared to the controls. In the cortical bone, the cortical thickness (*p* < 0.05) and cortical area (*p* < 0.05) were significantly lower and there was a tendency for the cortical BMD to be lower compared with the controls (*p* < 0.1). In the trabecular bone, there was a tendency for lower trabecular thickness (*p* < 0.1) and trabecular BMD (*p* < 0.1). In the G + L(2) female mice, there was a tendency for a decreased cortical thickness (*p* < 0.1), periosteal perimeter (*p* < 0.1), cortical area (*p* < 0.1), and cortical BMD (*p* < 0.1) compared with the controls. In the trabecular bone, the G + L(2) female mice had greater TMD (*p* < 0.01) and a tendency for decreased BMD (*p* < 0.1) compared with the controls. In the male offspring, the L(2) FLX group had a lower periosteal perimeter (*p* < 0.05) and cortical area (*p* < 0.05) compared with the control male offspring, but there were no significant differences in the trabecular parameters. Furthermore, there were no significant differences in the G + L(2) FLX mice in the cortical bone compared with the controls, but there was a tendency for a higher trabecular BMD in the G + L(2) mice compared with the control males (*p* < 0.1). There was, however, greater TMD (*p* < 0.01) and connectivity density (*p* < 0.01) in the trabecular bone. When comparing the two treatment groups, there were significant differences in the trabecular bone in both the female and male offspring. The G + L(2) females had a greater TMD and a lower connectivity density compared with the L(2) group. Further, there was a tendency for the G + L(2) females to have an increased trabecular number but reduced trabecular spacing than the L(2) group. In the males, the G + L(2) group had a significantly increased trabecular number, less trabecular spacing, greater TMD, and increased connectivity density than the L(2) group. There was also a tendency for the G + L(2) group to have greater trabecular thickness than the L(2) males.

**TABLE 2:** MicroCT was used to evaluate female cortical and trabecular bone parameters in mice exposed to 2 mg/kg fluoxetine during lactation or gestation and lactation at weaning.

	Female						
	CTRL	L(2) FLX	G + L(2) FLX	*p*‐value			
				One‐way ANOVA	CTRL vs L(2) FLX	CTRL vs G + L(2) FLX	L(2) FLX vs G + L(2) FLX
Cortical							
Ct.Th (mm)	0.072 ± 0.072	0.063 ± 0.006	0.064 ± 0.009	0.0396	0.0481	0.0933	0.9616
Ps.Pm (mm)	0.687 ± 0.024	0.653 ± 0.029	0.650 ± 0.043	0.0616	0.1067	0.0850	0.9817
BMD (mg Hg/cm^3^)	262.5 ± 32.38	232.7 ± 23.93	227.9 ± 27.67	0.0435	0.0952	0.0544	0.9340
TMD (mg Hg/cm^3^)	1090 ± 37.38	1053 ± 17.24	1059 ± 27.63	0.0312	0.8076	0.7594	0.9116
Trabecular							
Tb.N (1/mm)	4.112 ± 0.657	3.616 ± 0.354	4.197 ± 0.436	0.0633	0.1407	0.9379	0.0739
Tb.Sp (mm)	0.254 ± 0.041	0.283 ± 0.028	0.244 ± 0.028	0.0555	0.1474	0.8838	0.0597
Tb.Th (mm)	0.026 ± 0.002	0.024 ± 0.002	0.026 ± 0.002	0.0426	0.0502	>0.9999	0.1884
BMD (mg Hg/cm^3^)	79.14 ± 13.97	64.27 ± 11.43	84.85 ± 11.43	0.0090	0.0623	0.6311	0.0084
TMD (mg Hg/cm^3^)	867.7 ± 17.13	871.1 ± 9.750	896.3 ± 18.07	0.0023	0.8987	0.0035	0.0097
Conn.D (1/mm^3^)	139.3 ± 63.62	232.5 ± 80.44	85.47 ± 43.88	0.0063	0.3348	0.3348	0.0044

*Note*: Trabecular parameters of the female control (*n* = 8), L(2) FLX (*n* = 8), and G + L(2) FLX (*n* = 8) offspring and cortical parameters of the female control (*n* = 8), L(2) FLX (*n* = 9), and G + L(2) FLX (*n* = 8) offspring were measured. Data are presented as mean ± SD and analyzed using one‐way ANOVA for normally distributed data and a Kruskal–Wallis test for data that were not normally distributed; *p* < 0.05 is considered significant and 0.1 < *p* < 0.05 is considered a tendency.

Abbreviations: BMD, bone mineral density; Conn.D, connectivity density; Ct.Th, cortical thickness; Ps.Pm, periosteal perimeter; Tb.N., trabecular number; Tb.Sp., trabecular spacing; Tb.Th., trabecular thickness; TMD, tissue mineral density.

**TABLE 3:** MicroCT was used to evaluate male cortical and trabecular bone parameters in mice exposed to 2 mg/kg fluoxetine during lactation or gestation and lactation at weaning.

	Male						
	CTRL	L(2) FLX	G + L(2) FLX	*p*‐value			
				One‐way ANOVA	CTRL vs L(2) FLX	CTRL vs G + L(2) FLX	L(2) FLX vs G + L(2) FLX
Cortical							
Ct.Th (mm)	0.070 ± 0.006	0.063 ± 0.008	0.068 ± 0.008	0.1742	0.1868	0.9275	0.3447
Ps.Pm (mm)	0.690 ± 0.046	0.643 ± 0.026	0.6633 ± 0.034	0.0554	0.0442	0.3531	0.5124
BMD (mg Hg/cm^3^)	241.5 ± 19.21	240.7 ± 32.34	240.5 ± 23.69	0.9972	0.9981	0.9973	>0.9999
TMD (mg Hg/cm^3^)	1076 ± 18.44	1069 ± 26.39	1069 ± 26.72	0.7446	0.8076	0.7594	0.9938
Trabecular							
Tb.N (1/mm)	4.171 ± 0.508	3.769 ± 0.404	4.573 ± 0.246	0.0014	0.1002	0.1242	0.0009
Tb.Sp (mm)	0.247 ± 0.030	0.269 ± 0.032	0.221 ± 0.011	0.0034	0.1780	0.1402	0.0024
Tb.Th (mm)	0.026 ± 0.002	0.024 ± 0.001	0.026 ± 0.002	0.0517	0.1512	0.8543	0.0585
BMD (mg Hg/cm^3^)	77.21 ± 13.54	65.78 ± 12.86	93.53 ± 16.52	0.0017	0.2087	0.0668	0.0011
TMD (mg Hg/cm^3^)	868.4 ± 13.30	861.8 ± 10.39	892.2 ± 20.01	0.0009	0.6224	0.0083	0.0009
Conn.D (1/mm^3^)	152.7 ± 71.71	95.74 ± 62.62	271.7 ± 94.51	0.0002	0.1161	0.0074	0.0002

*Note*: Trabecular parameters of the male control (*n* = 9), L(2) FLX (*n* = 10), and G + L(2) FLX (*n* = 8) offspring and cortical parameters of the male control (*n* = 8), L(2) FLX (*n* = 10), and G + L(2) FLX (*n* = 8) offspring were measured. Data are presented as mean ± SD and analyzed using one‐way ANOVA for normally distributed data and a Kruskal–Wallis test for data that were not normally distributed; *p* < 0.05 is considered significant and 0.1 < *p* < 0.05 is considered a tendency.

Abbreviations: BMD, bone mineral density; Conn.D, connectivity density; Ct.Th, cortical thickness; Ps.Pm, periosteal perimeter; Tb.N., trabecular number; Tb.Sp., trabecular spacing; Tb.Th., trabecular thickness; TMD, tissue mineral density.

### 3.3 Exposure to a low or high dose of fluoxetine during lactation impacted cortical and trabecular bone in a sex‐dependent manner at weaning

**FIGURE 4:**
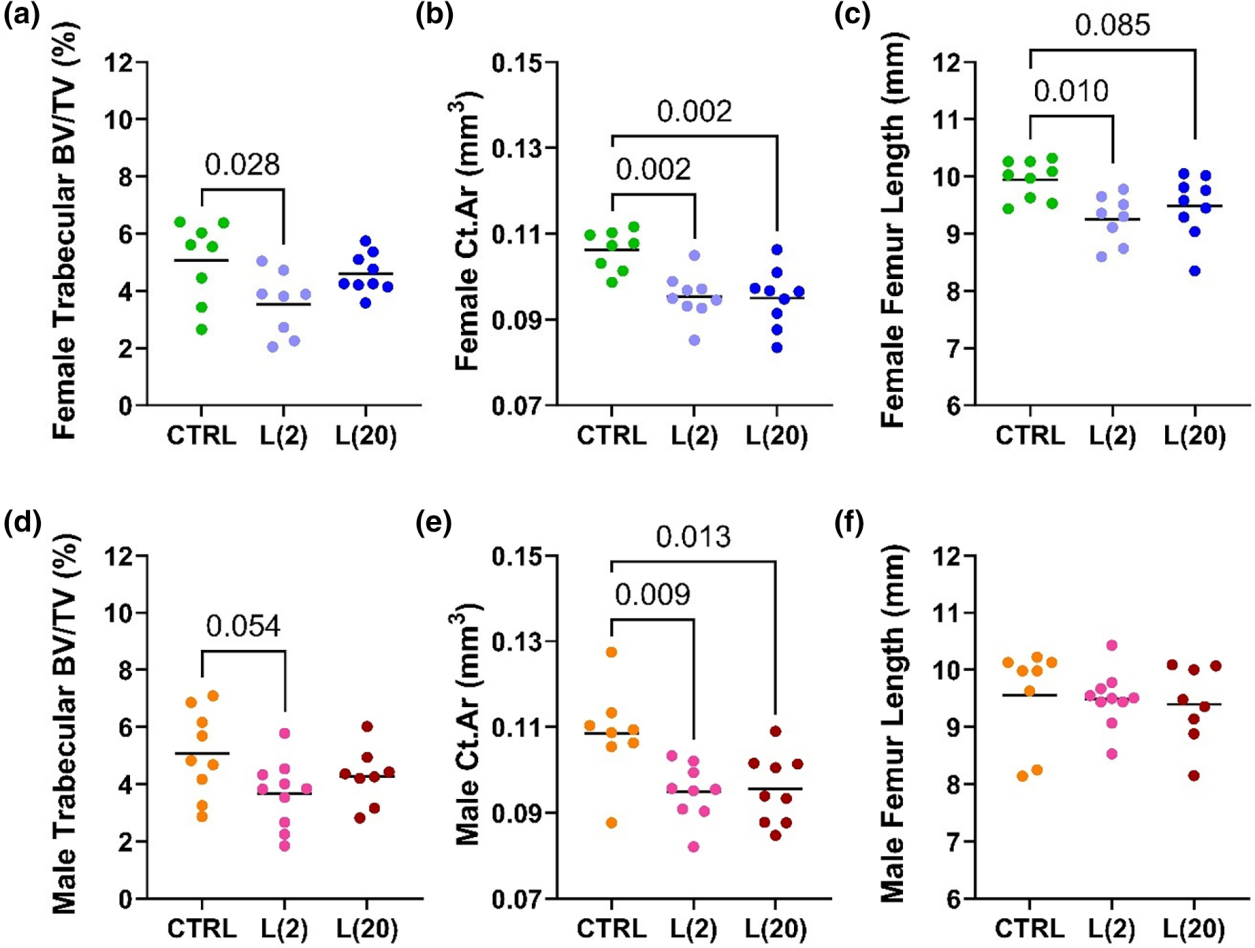
Fluoxetine exposure during lactation has dose‐dependent and sex‐specific impacts on cortical and trabecular characteristics at weaning. C57BL/6J dams were administered either 2 or 20 mg/kg fluoxetine during lactation. MicroCT was used to measure trabecular (control: *n* = 8 female, *n* = 9 male); L(2) FLX: *n* = 8 female, *n* = 10 male; and L(20) FLX: *n* = 9 female, *n* = 8 male) and cortical (control: *n* = 8 female, *n* = 8 male; L(2) FLX: *n* = 9 female, *n* = 10 male, and L(20) FLX: *n* = 9 female; *n* = 9 male) parameters in the female and male offspring. The trabecular BV/TV (a,d), cortical area (b,e), and femur length (c,f) were measured. Bars represent the mean; *p* < 0.05 is considered significant and 0.1 < *p* < 0.05 is considered a tendency.

In order to explore the effect of dose on lactational exposure to fluoxetine, MicroCT analysis was used to measure the cortical and trabecular parameters of exposure to either 2 mg/kg or 20 mg/kg of fluoxetine during lactation. In the female L(2) FLX group, the BV/TV was significantly lower (3.544% ± 1.105%) compared with the control (5.064% ± 1.408%; *p* < 0.05), but no differences were observed in the L(20) FLX group (4.600% ± 0.691%) (Figure 4a). This trend is recapitulated in the male L(2) FLX group, which has a tendency to have a lower BV/TV (3.663% ± 1.164%) compared with the control group (5.067% ± 1.501%; *p* < 0.1), but no difference in the L(20) group (4.274% ± 0.992%) (Figure 4d). In the female mice, the cortical area of both the L(2) FLX group (0.095 ± 0.005 mm^2^; *p* < 0.01) and the L(20) group (0.095 ± 0.007 mm^2^; *p* < 0.01) was significantly lower compared with the control mice (0.106 ± 0.005 mm^2^) (Figure 4b). Similarly, in the male offspring, both the L(2) group (0.109 ± 0.011 mm^2^; *p* < 0.01) and the L(20) group (0.096 ± 0.008 mm^2^; *p* < 0.05) had significantly lower cortical area than the control group (0.109 ± 0.011 mm^2^) (Figure 4e). The female L(2) FLX animals had shorter femurs (9.256 ± 0.418 mm) compared with the control group (9.948 ± 0.334 mm; *p* < 0.01), and the L(20) FLX tended to have shorter femurs (9.483 ± 0.539 mm) compared with the controls (*p* < 0.1) (Figure 4c); however, there were no significant differences in the male animals of either the L(2) FLX (9.492 ± 0.484 mm) or the L(20) FLX (9.396 ± 0.675 mm) compared with the control group (9.558 ± 0.860 mm) (Figure 4f).

In order to further investigate the skeletal characteristics in mice that were exposed to either 2 mg/kg or 20 mg/kg FLX during lactation, MicroCT was used to examine cortical and trabecular parameters of female (Table 4) and male (Table 5) offspring. Compared with the control animals, the L(2) FLX females had reduced cortical thickness (*p* < 0.05), periosteal perimeter (*p* < 0.05), cortical area (*p* < 0.01), and TMD (*p* < 0.05). In the trabecular bone, the L(2) FLX females had decreased trabecular thickness (*p* < 0.05) and tended to have greater connectivity density (*p* < 0.1). The L(20) FLX females exhibited a lower periosteal perimeter (*p* < 0.01) and cortical area (*p* < 0.01) but had no significant differences in any trabecular parameters. In the male mice, the L(2) FLX group and the L(20) FLX group had a lower periosteal perimeter (*p* < 0.05 and *p* < 0.05, respectively) and cortical area (*p* < 0.01 and *p* < 0.05, respectively). There were no significant differences in any trabecular parameters in either the L(2) FLX group or L(20) FLX group compared with the controls. When comparing the differences between treatment groups, there were significant differences only in the trabecular bone between the female L(2) and L(20) groups. The L(20) females had significantly higher connectivity density than the L(2) group.

**TABLE 4:** MicroCT was used to evaluate female cortical and trabecular bone parameters in mice exposed to 2 mg/kg or 20 mg/kg fluoxetine during lactation at weaning.

	Female						
	CTRL	L(2) FLX	L(20) FLX	*p*‐value			
				One‐way ANOVA	CTRL vs L(2) FLX	CTRL vs L(20) FLX	L(2) FLX vs L(20) FLX
Cortical							
Ct.Th (mm)	0.072 ± 0.008	0.063 ± 0.009	0.066 ± 0.006	0.0509	0.0421	0.2451	0.6066
Ps.Pm (mm)	0.687 ± 0.024	0.653 ± 0.027	0.649 ± 0.017	0.0064	0.0184	0.0097	0.9550
BMD (mg Hg/cm^3^)	262.5 ± 32.38	232.7 ± 23.93	250.9 ± 20.46	0.0745	0.0643	0.6275	0.3107
TMD (mg Hg/cm^3^)	1090 ± 37.38	1053 ± 17.24	1077 ± 27.49	0.0378	0.0334	0.6196	0.1904
Trabecular							
Tb.N (1/mm)	4.112 ± 0.657	3.616 ± 0.354	3.954 ± 0.308	0.1116	0.1022	0.7625	0.3060
Tb.Sp (mm)	0.251 ± 0.041	0.283 ± 0.028	0.254 ± 0.018	0.0746	0.1028	0.9870	0.1220
Tb.Th (mm)	0.026 ± 0.002	0.024 ± 0.002	0.025 ± 0.001	0.0324	0.0265	0.5825	0.4885
BMD (mg Hg/cm^3^)	79.14 ± 13.97	64.27 ± 11.43	75.43 ± 6.220	0.0307	0.0307	0.7637	0.1093
TMD (mg Hg/cm^3^)	867.7 ± 17.13	871.1 ± 9.750	871.7 ± 14.19	0.8202	0.8788	0.8231	0.9948
Conn.D (1/mm^3^)	139.3 ± 63.62	85.47 ± 43.88	152.6 ± 19.35	0.0195	0.0573	0.5710	0.0245

*Note*: Trabecular parameters of the female control (*n* = 8), L(2) FLX (*n* = 8), and L(20) FLX (*n* = 9) offspring and cortical parameters of the female control (*n* = 8), L(2) FLX (*n* = 9), and L(20) FLX (*n* = 9) offspring were measured. Data are presented as mean ± SD and analyzed using one‐way ANOVA for normally distributed data and a Kruskal–Wallis test for data that were not normally distributed; *p* < 0.05 is considered significant and 0.1 < *p* < 0.05 is considered a tendency.

Abbreviations: BMD, bone mineral density; Conn.D, connectivity density; Ct.Th, cortical thickness; Ps.Pm, periosteal perimeter; Tb.N., trabecular number; Tb.Sp., trabecular spacing; Tb.Th., trabecular thickness; TMD, tissue mineral density.

**TABLE 5:** MicroCT was used to evaluate male cortical and trabecular bone parameters in mice exposed to 2 mg/kg or 20 mg/kg fluoxetine during lactation at weaning.

	Male						
	CTRL	L(2) FLX	L(20) FLX	*p*‐value			
				One‐way ANOVA	CTRL vs L(2) FLX	CTRL vs L(20) FLX	L(2) FLX vs L(20) FLX
Cortical							
Ct.Th (mm)	0.070 ± 0.006	0.063 ± 0.008	0.065 ± 0.009	0.2307	0.2084	0.5026	0.8455
Ps.Pm (mm)	0.690 ± 0.046	0.643 ± 0.026	0.6476 ± 0.028	0.0178	0.0237	0.0450	0.9498
BMD (mg Hg/cm^3^)	241.5 ± 19.21	240.7 ± 32.34	238.8 ± 35.09	0.9826	0.9986	0.9823	0.9903
TMD (mg Hg/cm^3^)	1076 ± 18.44	1069 ± 26.39	1062 ± 28.23	0.5159	0.8170	0.4841	0.8342
Trabecular							
Tb.N (1/mm)	4.171 ± 0.508	3.769 ± 0.404	3.954 ± 0.330	0.1400	0.1182	0.5510	0.6317
Tb.Sp (mm)	0.247 ± 0.030	0.269 ± 0.032	0.257 ± 0.024	0.2556	0.2288	0.7669	0.6310
Tb.Th (mm)	0.026 ± 0.002	0.024 ± 0.001	0.025 ± 0.001	0.1157	0.1095	0.8284	0.3369
BMD (mg Hg/cm^3^)	77.21 ± 13.54	65.78 ± 12.86	71.16 ± 9.609	0.1491	0.1264	0.5740	0.6294
TMD (mg Hg/cm^3^)	868.4 ± 13.30	861.8 ± 10.39	871.5 ± 7.507	0.1857	0.4133	0.8239	0.1761
Conn.D (1/mm^3^)	152.7 ± 71.71	95.74 ± 62.62	107.5 ± 54.70	0.1498	0.1781	0.2890	0.7009

*Note*: Trabecular parameters of the male control (*n* = 9), L(2) FLX (*n* = 10), and L(20) FLX (*n* = 8) offspring and cortical parameters of the male control (*n* = 8), L(2) FLX (*n* = 9), and L(20) FLX (*n* = 9) offspring were measured. Data are presented as mean ± SD and analyzed using one‐way ANOVA for normally distributed data and a Kruskal–Wallis test for data that was not normally distributed; *p* < 0.05 is considered significant and 0.1 < *p* < 0.05 is considered a tendency.

Abbreviations: BMD, bone mineral density; Conn.D, connectivity density; Ct.Th, cortical thickness; Ps.Pm, periosteal perimeter; Tb.N., trabecular number; Tb.Sp., trabecular spacing; Tb.Th., trabecular thickness; TMD, tissue mineral density.

### 3.4 Fluoxetine exposure during lactation alone has long‐term implications on bone that are minimally recapitulated in offspring exposed during both gestation and lactation

**FIGURE 5:**
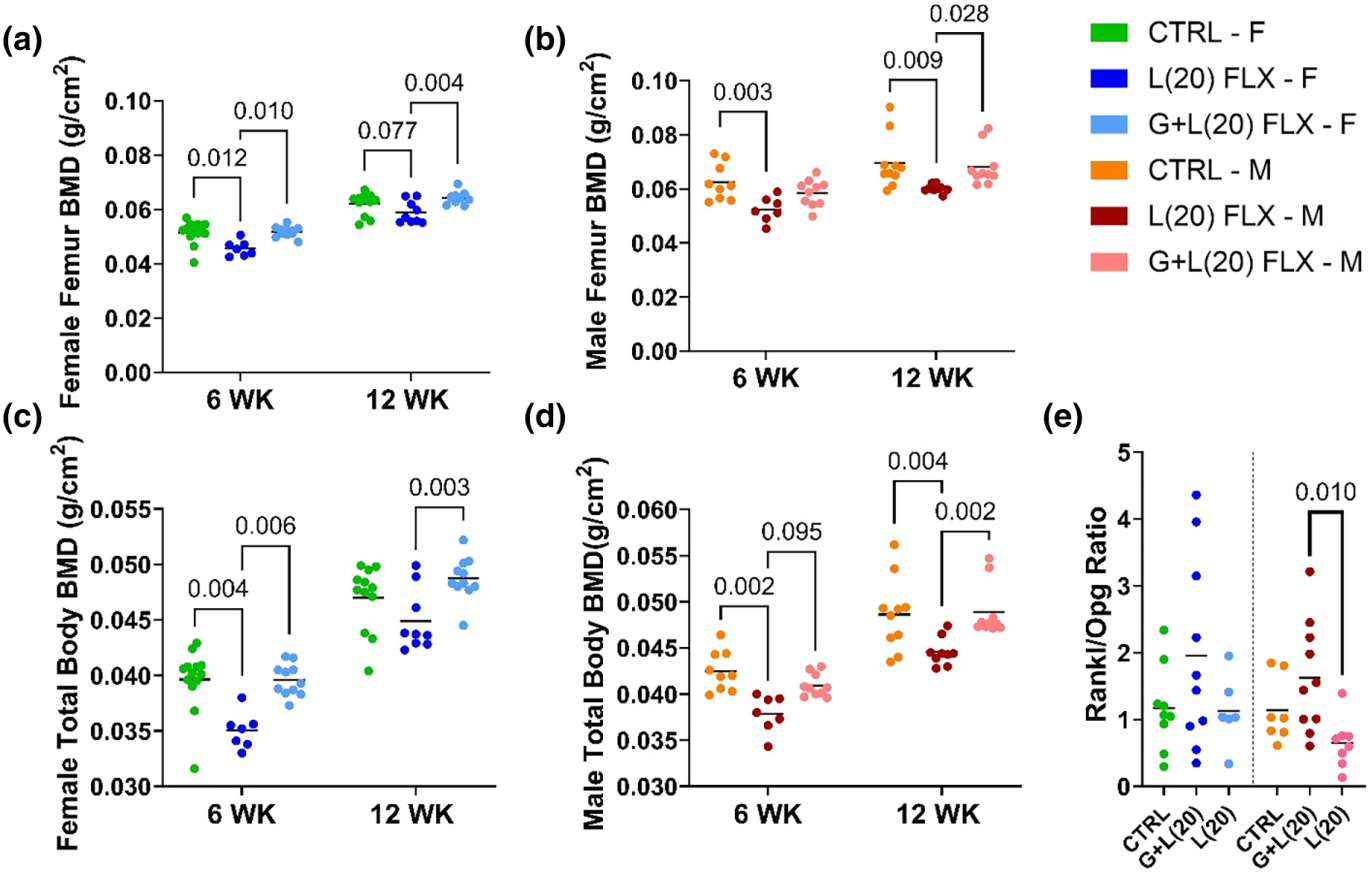
Developmental fluoxetine exposure has differential effects on adult skeletal characteristics depending on the window of exposure and the sex of the offspring. C57BL/6J dams were administered 20 mg/kg fluoxetine daily either during lactation or the entire peripartum period. The exposed offspring were then aged out to 12 weeks of age before their femurs were collected. Dual‐energy x‐ray absorptiometry (DEXA) was used to measure bone mineral density (BMD) of the femur of the female (a) offspring (*n* = 13 CTRL; *n* = 9 L(20); *n* = 11 G + L(20)) and male (b) offspring (*n* = 10 CTRL; *n* = 9 L(2); *n* = 10 L(20)), and the total body BMD was also measured via DEXA in the female (c) and male (d) offspring at 6 and 12 weeks of age. Once femurs were collected from the offspring, RNA was extracted, and the ratio of RANKL/OPG mRNA relative expression was determined in the female and male offspring (e). Bars represent the mean; *p* < 0.05 is considered significant and 0.1 < *p* < 0.05 is considered a tendency.

To determine the phenotypic effects of developmental fluoxetine exposure on adult bone, DEXA was used to measure the BMD of the femur and the total body at 6 and 12 weeks of age. In the female offspring, the L(20) group had a lower BMD of the femur than the control group and the G + L(20) group at 6 weeks of age (*p* < 0.05 and *p* < 0.01, respectively). There was a tendency for the L(2) group to have a lower femoral BMD at 12 weeks of age compared with the control group (*p* < 0.1), and significantly lower BMD compared with the G + L(20) animals (*p* < 0.01) (Figure 5a). In the male offspring, the L(20) mice had a lower femoral BMD at 6 weeks of age compared with the control group (*p* < 0.01), and at 12 weeks of age, had a significantly lower BMD compared with the control group (*p* < 0.01) and the G + L(20) group (*p* < 0.05) (Figure 5b). A similar pattern was observed in the total body BMD. In the female mice, there was lower BMD at 6 weeks of age compared with the control group (*p* < 0.01) and the G + L(20) group (*p* < 0.01). At 12 weeks of age, there was significantly lower BMD of the L(20) group compared with the G + L(20) group (*p* < 0.01) (Figure 5c). In the males, the L(20) group had a decreased total body BMD compared with the controls (*p* < 0.01) and a tendency to have lower BMD compared with the G + L(20) group (*p* < 0.1) at 6 weeks of age. At 12 weeks of age, the L(20) group had lower BMD compared with the control group (*p* < 0.01) and the G + L(20) group (*p* < 0.01) (Figure 5d). The long‐term impact of developmental fluoxetine exposure was further explored via relative gene expression in the femur. First, the RANKL/OPG ratio of relative gene expression, a measurement of the ratio of bone breakdown to bone building, was examined in the female and male offspring. In the female offspring, there were no significant differences between any of the groups (Figure 5e). In the male offspring, the L(20) group had a lower RANKL/OPG ratio compared with the G + L(20) group (*p* < 0.01).

**FIGURE 6:**
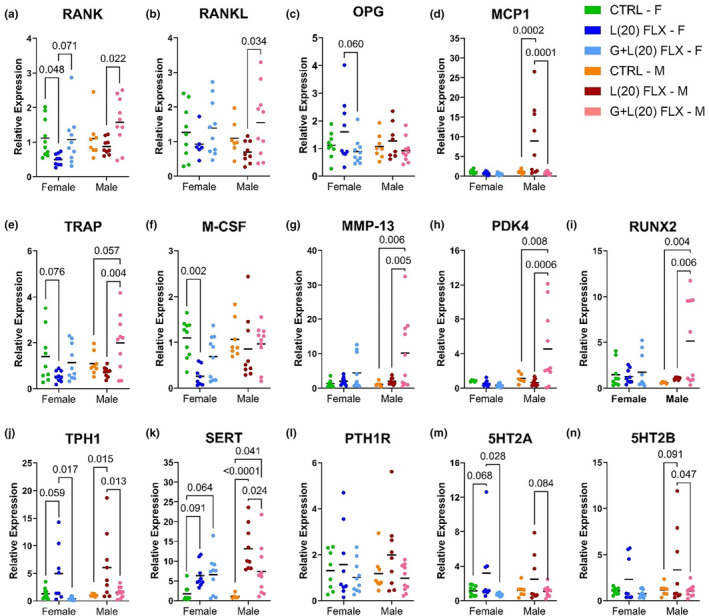
Relative gene expression of the femur of female and male offspring. At 12 weeks old, the femur was collected from the female offspring (*n* = 13 CTRL; *n* = 9 L(20); *n* = 11 G + L(20)) and male offspring (*n* = 10 CTRL; *n* = 9 L(2); *n* = 10 L(20)) and gene expression was quantified via RT‐PCR. The relative gene expression of *Rank* (a), *Rankl* (b), *Opg* (c), *Mcp1* (d), *Trap* (e), *M‐csf* (f), *Mmp13* (g), *Pdk4* (h), *Runx2* (i), *Tph1* (j), *Sert* (k), *Pth1r* (l), *5ht2a* (m), and *5ht2b* (n) were measured. Bars represent the mean; *p* < 0.05 is considered significant and 0.1 < *p* < 0.05 is considered a tendency.

The relative gene expression was further investigated in the femurs of female and male offspring (Figure 6a–n). In the female mice, there was a tendency for there to be lower *Rank* expression in the L(20) group compared with the control and G + L(20) group (*p* < 0.1 and *p* < 0.1, respectively), but the male L(20) group had significantly lower expression of *Rank* compared with the G + L(20) group (*p* < 0.05). Similarly, in the male mice, there was also lower *Rankl* expression in the L(20) group compared with the G + L(20) group (*p* < 0.05), but no difference in the female mice. There was a tendency for *Opg* expression to be greater in the L(20) offspring compared with the G + L(20) offspring (*p* < 0.1), but there were no significant differences between any of the groups of either sex. In the L(20) females, there was a tendency for *Trap* expression to be lower compared with the controls (*p* < 0.1), as well as a tendency for the G + L(20) males to be greater compared with the controls (*p* < 0.1) and was significantly greater compared with the L(20) group. The relative expression of *M‐csf* was downregulated in the G + L(20) females compared with the controls (*p* < 0.05), and in the males, *Mmp13* expression was upregulated in the L(20) group compared with the controls and the G + L(20) group (*p* < 0.01 and *p* < 0.01, respectively). In the male mice, *Pdk4* expression was upregulated in the G + L(20) group compared with both the control (*p* < 0.01) and L(20) (*p* < 0.001) groups. Similarly, *Runx2* expression was also lower in the control and L(20) groups compared with the G + L(20) group (*p* < 0.05 and *p* < 0.05, respectively). In the female mice, there was a tendency for *Tph1* to be upregulated in the L(20) mice compared with the controls (*p* < 0.1) and was significantly upregulated compared with the G + L(20) offspring (*p* < 0.05). In the male mice, *Tph1* was upregulated in the L(20) offspring compared with both the control (*p* < 0.05) and the G + L(20) offspring (*p* < 0.05). There was no significant difference in *Sert* expression in the female offspring, but there was a tendency for both the L(20) and G + L(20) to be greater compared with the control group (*p* < 0.1 and *p* < 0.1, respectively). In the male offspring, there was greater *Sert* expression in the L(20) offspring compared with the controls (*p* < 0.0001) and the G + L(20) offspring (*p* < 0.05) and was also significantly greater in the G + L(20) compared with the control group (*p* < 0.05). There were no significant differences in the expression of *Pth1r* in either sex among treatment groups. In the female offspring, there was a tendency for there to be an upregulation of *5ht2a* expression in the L(20) group compared with the controls (*p* < 0.1) and a significant upregulation compared with the G + L(20) group (*p* < 0.05). In the male mice, there was a tendency for *5ht2a* expression to be upregulated in the L(20) group compared with the G + L(20) group (*p* < 0.1), but there were no significant differences. Finally, in the male offspring, there was a tendency for *5ht2b* expression to be upregulated in the L(20) group compared with the control animals (*p* < 0.1), and a significant upregulation of *5ht2b* expression compared with the G + L(20) group (*p* < 0.05).

MicroCT was used to further explore the long‐term effects of developmental exposure to 20 mg/kg fluoxetine on the trabecular and cortical characteristics of both female (Table 6) and male (Table 7) offspring. In the female mice, there was a tendency for the G + L(20) FLX mice to have shorter femurs compared with the control group (*p* < 0.1), but there were no other significant differences in either the cortical or trabecular parameters of the L(20) FLX or G + L(20) FLX groups compared with the control group. In the male mice, there was a significant decrease in TMD in the L(20) FLX group compared with the controls (*p* < 0.05). In the trabecular bone, the L(20) FLX group had a tendency to have greater trabecular spacing (*p* < 0.1), TMD (*p* < 0.1), and lower connectivity density (*p* < 0.1) compared with the control group. In the male G + L(20) FLX animals, there were no significant differences in cortical or trabecular bone parameters compared with the control group. When comparing the G + L(20) and L(20) female offspring, there tended to be differences in the cortical and the trabecular bone. In the cortical bone, the G + L(20) females tended to have a greater periosteal perimeter and cortical area compared with the L(20) females. In the trabecular bone, there was a tendency for the G + L(20) females to have a greater trabecular thickness, TMD, and connectivity density compared with the L(20) animals. Between the male G + L(20) and L(20) groups, there was a tendency for there to be a greater trabecular number, TMD, and connectivity density and less trabecular spacing in the G + L(20) group compared with the L(20) group.

**TABLE 6:** MicroCT was used to evaluate female cortical and trabecular bone parameters in mice exposed to 20 mg/kg fluoxetine during lactation or gestation and lactation 12 weeks of age.

	Female						
	CTRL	L(20) FLX	G + L(20) FLX	*p*‐value			
				One‐way ANOVA	CTRL vs L(20) FLX	CTRL vs G + L(20) FLX	L(20) FLX vs G + L(20) FLX
Cortical							
Ct.Th (mm)	0.160 ± 0.015	0.153 ± 0.008	0.157 ± 0.007	0.3487	0.3159	0.8103	0.6692
Ps.Pm (mm)	8.146 ± 0.230	7.882 ± 0.309	8.245 ± 0.202	0.0430	0.1067	>0.9999	0.0624
Ct.Ar (mm^2^)	1.569 ± 0.056	1.478 ± 0.089	1.581 ± 0.053	0.0546	0.1049	>0.9999	0.0944
BMD (mg Hg/cm^3^)	532.8 ± 40.73	525.1 ± 22.32	528.1 ± 24.62	0.8477	0.8417	0.9303	0.9758
TMD (mg Hg/cm^3^)	1210 ± 7.765	1198 ± 19.75	1211 ± 14.21	0.0980	0.1516	0.9741	0.1183
Length (mm)	15.32 ± 0.166	15.21 ± 0.216	15.11 ± 0.231	0.0664	0.4294	0.0543	0.5833
Trabecular							
BV/TV (%)	6.126 ± 0.474	5.912 ± 1.425	6.621 ± 0.920	0.2508	0.8716	0.4481	0.2498
Tb.N (1/mm)	3.850 ± 0.208	3.835 ± 0.310	3.783 ± 0.176	0.7724	0.9880	0.7671	0.8709
Tb.Sp (mm)	0.258 ± 0.0145	0.260 ± 0.021	0.262 ± 0.013	0.8367	0.9496	0.8224	0.9650
Tb.Th (mm)	0.037 ± 0.005	0.034 ± 0.002	0.037 ± 0.002	0.0538	0.1652	>0.9999	0.0640
BMD (mg Hg/cm^3^)	47.51 ± 5.59	48.53 ± 18.81	53.34 ± 11.60	0.5091	0.9812	0.5103	0.6710
TMD (mg Hg/cm^3^)	984.1 ± 10.19	977.9 ± 9.132	988.7 ± 10.78	0.0754	0.3541	0.5068	0.0608
Conn.D (1/mm^3^)	134.0 ± 15.03	121.7 ± 36.86	146.1 ± 18.55	0.0951	0.4866	0.4594	0.0784

*Note*: Trabecular parameters of the female control (*n* = 13), L(20) FLX (*n* = 9), and G + L(20) FLX (*n* = 11) offspring and cortical parameters of the female control (*n* = 13), L(20) FLX (*n* = 9), and G + L(20) FLX (*n* = 11) offspring were measured. Data are presented as mean ± SD and analyzed using one‐way ANOVA for normally distributed data and a Kruskal–Wallis test for data that were not normally distributed; *p* < 0.05 is considered significant and 0.1 < *p* < 0.05 is considered a tendency.

Abbreviations: BMD, bone mineral density; BV/TV, bone volume fraction; Conn.D, connectivity density; Ct.Ar, cortical area; Ct.Th, cortical thickness; Ps.Pm, periosteal perimeter; Tb.N., trabecular number; Tb.Sp., trabecular spacing; Tb.Th., trabecular thickness; TMD, tissue mineral density.

**TABLE 7:** MicroCT was used to evaluate male cortical and trabecular bone parameters in mice exposed to 20 mg/kg fluoxetine during lactation or gestation and lactation at 12 weeks of age.

	Male						
	CTRL	L(20) FLX	G + L(20) FLX	*p*‐value			
				One‐way ANOVA	CTRL vs L(20) FLX	CTRL vs G + L(20) FLX	L(20) FLX vs G + L(20) FLX
Cortical							
Ct.Th (mm)	0.160 ± 0.016	0.150 ± 0.006	0.156 ± 0.013	0.2295	0.2016	0.7182	0.5428
Ps.Pm (mm)	8.990 ± 0.602	8.438 ± 0.318	8.942 ± 0.782	0.1442	0.1682	0.9834	0.2088
Ct.Ar (mm^2^)	1. 815 ± 0.198	1.631 ± 0.100	1.749 ± 0.217	0.1302	0.1116	0.6955	0.3738
BMD (mg Hg/cm^3^)	506.5 ± 34.84	493.5 ± 21.03	508.2 ± 31.52	0.5445	0.6395	0.9911	0.5539
TMD (mg Hg/cm^3^)	1200 ± 13.08	1183 ± 14.37	1193 ± 13.44	0.0345	0.0268	0.4706	0.2212
Length (mm)	15.25 ± 0.158	15.20 ± 0.216	15.02 ± 0.407	0.1959	0.9224	0.2010	0.4037
Trabecular							
BV/TV (%)	10.17 ± 3.686	8.440 ± 0.477	9.566 ± 0.804	0.3057	0.2783	0.8410	0.5846
Tb.N (1/mm)	4.820 ± 0.474	4.755 ± 0.135	5.007 ± 0.105	0.0195	0.2528	0.7224	0.0156
Tb.Sp (mm)	0.195 ± 0.010	0.204 ± 0.006	0.188 ± 0.012	0.0048	0.0960	0.9012	0.0035
Tb.Th (mm)	0.035 ± 0.005	0.033 ± 0.002	0.033 ± 0.001	0.5341	0.8272	>0.9999	>0.9999
BMD (mg Hg/cm^3^)	98.25 ± 42.69	82.59 ± 6.670	93.41 ± 10.34	0.4773	0.4531	0.9203	0.6925
TMD (mg Hg/cm^3^)	978.8 ± 12.81	966.9 ± 8.500	981.8 ± 11.26	0.0230	0.0827	0.8161	0.0216
Conn.D (1/mm^3^)	230.3 ± 59.93	201.9 ± 21.40	252.1 ± 35.95	0.0250	0.0952	>0.9999	0.0290

*Note*: Trabecular parameters of the male control (*n* = 10), L(20) FLX (*n* = 9), and G + L(20) FLX (*n* = 11) offspring and cortical parameters of the male control (*n* = 10), L(20) FLX (*n* = 9), and G + L(20) FLX (*n* = 11) offspring were measured. Data are presented as mean ± SD and analyzed using one‐way ANOVA for normally distributed data and a Kruskal–Wallis test for data that were not normally distributed; *p* < 0.05 is considered significant and 0.1 < *p* < 0.05 is considered a tendency.

Abbreviations: BMD, bone mineral density; BV/TV, bone volume fraction; Conn.D, connectivity density; Ct.Ar, cortical area; Ct.Th, cortical thickness; Ps.Pm, periosteal perimeter; Tb.N., trabecular number; Tb.Sp., trabecular spacing; Tb.Th., trabecular thickness; TMD, tissue mineral density.

### 3.5 Fluoxetine exposure during lactation only, but not both gestation and lactation, has long‐term effects on the body composition of adult offspring

**FIGURE 7:**
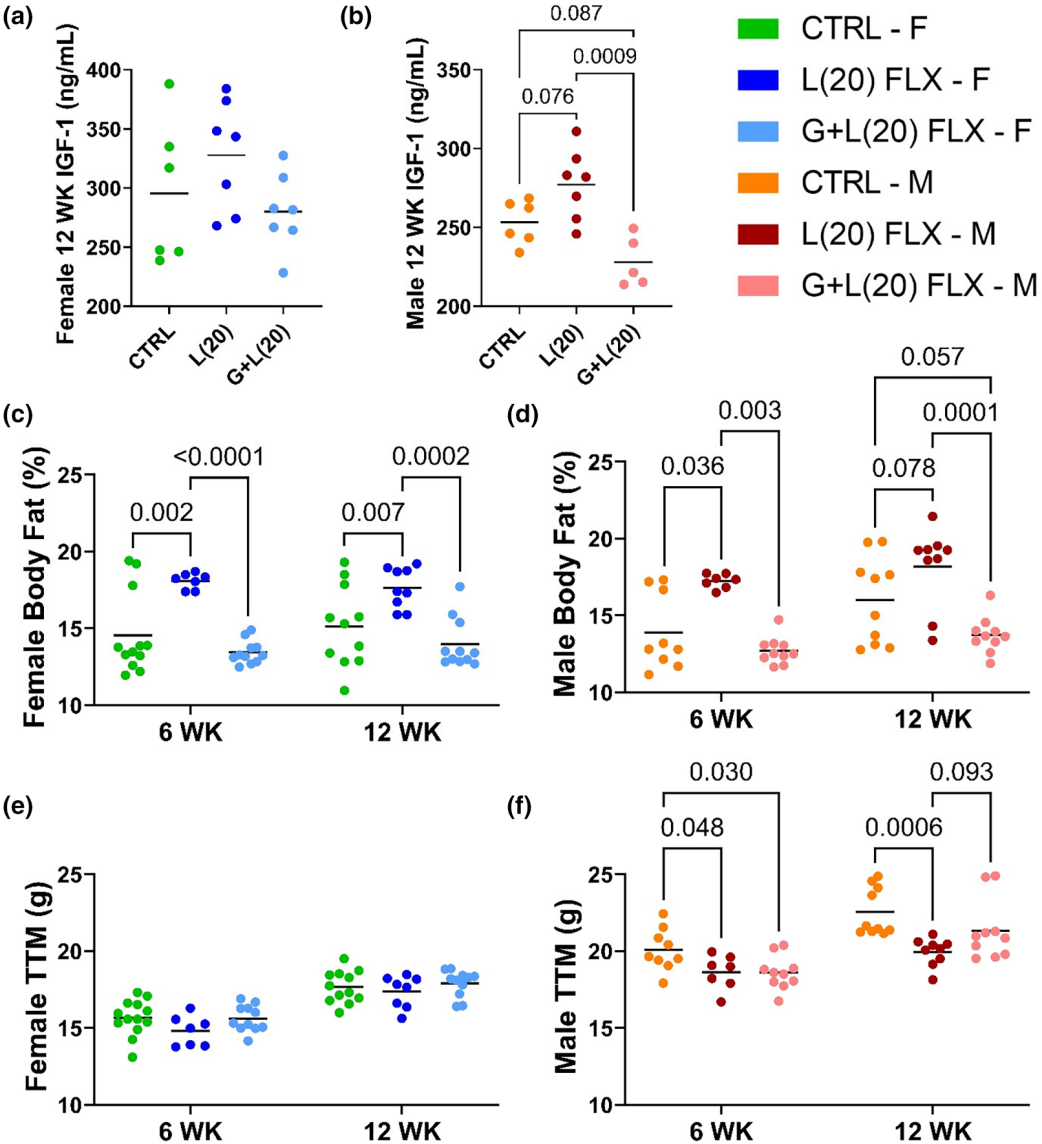
Exposure to a high dose of fluoxetine during development has differential effects on body composition dependent on the window and length of exposure. C57BL/6J dams were administered 20 mg/kg fluoxetine during lactation alone or throughout gestation and lactation. The offspring of these dams that were exposed to fluoxetine during development were then aged out to 12 weeks of age before their body composition was analyzed. Circulating IGF‐1 concentrations were measured in the female (a) and male (b) offspring. Dual‐energy x‐ray absorptiometry was used to measure the percent body fat of the female (c) and male (d) mice at 6 and 12 weeks of age and the total tissue mass of the female (e) and male (f) mice at the same time points. Bars represent the mean; *p* < 0.05 is considered significant and 0.1 < *p* < 0.05 is considered a tendency.

Circulating IGF‐1 was measured in the serum of the exposed offspring at 12 weeks of age. There were no significant differences in IGF‐1 concentrations in the female mice (Figure 7a). In the male offspring, there was a tendency for increased IGF‐1 concentrations in the L(20) FLX group (*p* < 0.1) and lower in the G + L(20) FLX group (*p* < 0.1). Circulating IGF‐1 concentrations were significantly lower in the male G + L(20) FLX compared with the L(20) FLX group (*p* < 0.001) (Figure 7b). Dual‐energy x‐ray absorptiometry (DEXA) was used to measure the percent body fat of both the female and male mice, as well as the total tissue mass (TTM) at 6 and 12 weeks of age. In the female mice, the percent body fat was greater in the L(20) FLX mice compared with both the control group and the G + L(20) FLX group at 6 weeks of age (*p* < 0.01 and *p* < 0.0001, respectively) and at 12 weeks of age (*p* < 0.01 and *p* < 0.001, respectively) (Figure 7c). Similarly, in the male exposed offspring, the percent body fat was greater in the L(20) FLX mice compared with both the control group and the G + L(20) FLX group at 6 weeks of age (*p* < 0.05 and *p* < 0.01, respectively) (Figure 7d). At 12 weeks of age, there was a tendency for the L(20) FLX group to be higher (*p* < 0.1) and for the G + L(20) FLX group to be lower (*p* < 0.1) than the control group. Furthermore, the L(20) FLX male mice had a higher percent body fat at 12 weeks of age compared with the G + L(20) FLX group (*p* < 0.001). In the female mice, there were no significant differences in TTM between any of the groups at either 6 or 12 weeks of age (Figure 7e). In the male offspring, there was a significant decrease in TTM in both fluoxetine‐exposed groups compared with the control group at 6 weeks of age (*p* < 0.05 and *p* < 0.05, respectively). At 12 weeks of age, the L(20) FLX group had lower TTM compared with the control group (*p* < 0.001). Furthermore, there was a trend for the G + L(20) FLX group to have a higher TTM compared with the L(20) FLX group (*p* < 0.1).

## 4 DISCUSSION

The results of these experiments provide evidence that fluoxetine exposure during critical periods of development has consequences on the offspring that may be perpetuated into adulthood in a sex‐dependent manner. In the 3‐week‐old offspring, there appeared to be both a time‐dependent effect of fluoxetine exposure on bone, as well as a dose‐dependent effect. In both experiments focused on the offspring at weaning, there was a negative impact on bone in the mice exposed to a low dose of fluoxetine during lactation only, and this effect was not as robust in the mice exposed during both gestation and lactation or the mice exposed to a high dose of fluoxetine during lactation only. Furthermore, there was a more dramatic effect in the female offspring as opposed to the males. In the adult offspring that had been exposed to a high dose of fluoxetine during development, to the results suggest that there may be a greater negative effect on the animals exposed during lactation only as opposed to gestation and lactation. Interestingly, we also discovered that there was an impact of fluoxetine exposure on adult body composition in the offspring. Similar to the offspring at weaning, there was a greater impact on body composition in the animals exposed during lactation only, but conversely to what was seen in the offspring at weaning, this effect appeared to be more pronounced in the male offspring.

Aside from paroxetine, SSRIs are generally considered safe to be used during the peripartum period and are thus the most prescribed antidepressant during this time period (Hendrick et al., 2003). Overall, the implications of SSRI exposure during development have been generally inconclusive, and a confounding factor in human studies is the relationship between the underlying maternal psychiatric conditions during the peripartum period and adverse perinatal outcomes in the offspring (di Scalea & Wisner, 2009; Heikkinen et al., 2003; Hendrick et al., 2003; Yoshida et al., 1998). Therefore, it is critical to turn to animal studies in order to further elucidate the consequences of SSRI exposure during development on the offspring. Using a rodent model, we investigated different doses of fluoxetine exposure, different windows of exposure, and both the short‐term and long‐term effects of exposure on the skeleton of the exposed animals.

Serotonin is responsible for behavioral and metabolic processes centrally, and many physiological processes peripherally, including vasoconstriction, bone remodeling, and lactation (Erspamer & Asero, 1952; Lucki, 1998; Margolis et al., 2014; Matsuda et al., 2004; Yadav et al., 2008). We demonstrated that in femurs of the adult exposed offspring, the nonneuronal form of the rate‐limiting enzyme in serotonin synthesis, tryptophan hydroxylase 1 (TPH1) mRNA expression was upregulated in both the male and female offspring exposed to a high dose of fluoxetine during lactation only, but this was not recapitulated in the animals exposed during both gestation and lactation (Figure 6k). This finding suggests that perhaps exposure to a high dose of fluoxetine during lactation, but not gestation and lactation, results in a long‐term upregulation of serotonin synthesis in bone. This may be due, in part, to an adaptation of the bone in the offspring exposed to SSRI during both pregnancy and lactation that is not observed in the animals exposed postnatally only.

The serotonin transporter, which is encoded by the gene *SLC6A4*, is present both peripherally and centrally and is genetically identical throughout the body. In this study, we chose fluoxetine to examine the effects of developmental exposure to SSRIs for a few reasons. Firstly, fluoxetine was the first SSRI introduced in the United States and remains widely popular to this day (Fuentes et al., 2018; Wong et al., 2005). The doses of fluoxetine were chosen because they represent the upper and lower ranges of circulating fluoxetine in the human population (Dulawa et al., 2004). In the dams, we have previously demonstrated that administration of 20 mg/kg fluoxetine during the peripartum period has negative consequences on the maternal skeleton that are not restored post‐weaning (Weaver et al., 2018). Furthermore, we have reported that, at weaning, mice that were exposed to 20 mg/kg fluoxetine throughout gestation and lactation exhibited lower trabecular and cortical BMD, lower trabecular BV/TV, and shorter femurs (Weaver et al., 2019). When compared to the offspring exposed to the low dose of fluoxetine during both gestation and lactation, both the female and male mice exposed to the low dose during lactation had lower trabecular BV/TV (Figure 3a,b). However, while both sexes exposed to the low dose during lactation had lower trabecular BV/TV compared with the control animals, this difference was not recapitulated in the offspring exposed to a high dose of fluoxetine during the same time frame (Figure 4a,b). Modulation of bone remodeling by serotonin was first demonstrated over two decades ago by two independent studies: one reported that both SERT and serotonin receptors were present on osteoblasts, while the other revealed that the 5‐HT_2B_ receptor was present in fetal chick bone tissue and murine osteoblast cultures (Bliziotes et al., 2001; Weissman et al., 2004). However, in the female adult exposed offspring, there were no differences in 5‐HT_2B_ expression in the femur of either fluoxetine‐exposed groups, but there was a significant upregulation of 5‐HT_2B_ expression in the lactation‐only male mice compared with the mice exposed during the entire peripartum period.

Interestingly, in the aforementioned study, Weaver et al. did not observe sex differences that were demonstrated in the present study; however, the study was much smaller and only used the 20 mg/kg fluoxetine dose throughout gestation and lactation (Weaver et al., 2019). The female animals that were exposed to the low dose of fluoxetine during either lactation or the entire peripartum period had shorter femurs, and while there was a tendency for the lactation‐only high dose animals to have shorter femurs, the difference was not significant. In the male mice, there were no differences in femur length, suggesting a relationship between sex and the degree of impact developmental fluoxetine has on the adolescent skeleton. There is an established relationship between serotonin and ovarian hormones that might help clarify the mechanism of action behind the sexually dimorphic skeletal phenotypes observed in this study. Estrogen acts on its receptors present on serotonergic cells to reduce neurite growth, and progestin receptors are present on serotonin neurons, which suggests that ovarian hormones may influence the central regulation of bone remodeling (Bethea, 1993; Lu et al., 2004).

One of the more unexpected findings of this study was the changes in body composition exhibited in the adult offspring. Circulating IGF‐1 concentrations were unchanged in the female mice, but there were variations in the male mice, with the animals exposed to fluoxetine during lactation only having greater IGF‐1 concentrations compared with the animals exposed during the entire peripartum period. Growth hormone (GH) is responsible for the release of IGF‐1 from the liver, and at the level of the bone, IGF‐1 facilitates endochondral ossification and bone turnover (Bianda et al., 1997; Guntur & Rosen, 2013). In a study conducted in infants, Javaid et al. demonstrated a positive association between umbilical cord IGF‐1 concentrations and proportionate fat mass, and a negative association with proportional lean mass at birth, which is consistent with our results in the male fluoxetine‐exposed adult offspring; the lactation‐only mice had greater circulating IGF‐1 concentrations and higher percent body fat, while the circulating IGF‐1 concentrations and percent body fat of the mice exposed during both gestation and lactation did not differ significantly from the controls (Javaid et al., 2004). The female exposed offspring did not have differing concentrations of circulating IGF‐1 between any groups and yet phenotypically exhibited a greater percent body fat in the animals exposed during lactation only, but not those exposed through the entire peripartum period. An interesting note is that IGF‐1 cannot cross the placenta, and so umbilical cord IGF‐1 concentrations are primarily determined by the IGF‐1 production by the fetal liver (Javaid et al., 2004). The differences seen in IGF‐1 concentrations exhibited in the adult offspring may be attributed to the sexually dimorphic characteristics of the serotonergic system of the offspring during the peripartum period. The neurological and behavioral outcomes of developmental perturbation of the serotonergic system has been extensively studied, and there is evidence of sexually dimorphic effects of developmental antidepressant exposure in these contexts; however, the possible effect of sex steroids on antidepressant exposure during development in the context of adult body composition has yet to be elucidated (Campbell et al., 2021; Pawluski & Gemmel, 2018).

Overall, the mice that were exposed to fluoxetine during the lactational period only had a more profound effect on adverse skeletal phenotypes and body composition both at weaning and into adulthood. The idea of fetal programming, or the fetal origins hypothesis, postulates that there are factors in fetal and early postnatal life that can have ramifications on adult health (Barker, 1990). It is thought that the primary mechanism of fetal programming is epigenetic modification of gene expression via processes that include DNA methylation, micro‐RNA expression, and histone modification (Gluckman et al., 2008). The epigenetic mechanisms of fetal programming are still being elucidated. Recently, it has been demonstrated that monoamines such as serotonin can participate in epigenetic regulation via monoaminylation, and in the case of serotonin specifically, serotonylation (Walther et al., 2011). In the present study, the animals that were exposed during both gestation and lactation as opposed to lactation alone appeared to be more resilient to the skeletal effects of developmental fluoxetine exposure, and it is possible that fetal exposure to fluoxetine programmed the offspring to have a resistance to the drug exposure that was not recapitulated in the lactation‐only animals. Interestingly, there appeared to be a greater effect of lactational exposure to the low dose of fluoxetine in the offspring at weaning than the high dose. It has been proposed that in the case of indirect environmental exposure, fluoxetine exhibits a non‐monotonic, or biphasic, dose–response curve, in which low doses are stimulatory, while high doses are inhibitory (Bossus et al., 2014; Munari et al., 2014). Perhaps this nonlinear dose–response phenomenon can explain why skeletal phenotypes were more significantly impacted in the offspring exposed to a low dose of fluoxetine, but not a high dose.

In conclusion, exposure to a low dose of fluoxetine during lactation may have significant short‐term consequences on the skeleton of the offspring, while this effect is less dramatic in the offspring exposed to either the high dose of fluoxetine during lactation or the low dose during both gestation and lactation. Furthermore, exposure to a high dose of fluoxetine during lactation may have an impact on adult body composition, while animals exposed during the entire peripartum period may be protected from these effects. There are several limitations to this study that are important to discuss. Firstly, we did not examine the effects of fluoxetine exposure during gestation alone, and this is primarily due to the issues with pregnancy loss and pup mortality we have observed in the past, especially in the high dose of fluoxetine (Domingues et al., 2022). Furthermore, we did not examine the long‐term effect of exposure to the low dose of fluoxetine on the adult skeleton, as we did not expect to see a greater impact on bone parameters in the animals exposed to a low dose of fluoxetine as opposed to the high dose. Future work should be done to further investigate the possible protective effect of fetal programming in response to gestational fluoxetine exposure, as well as the long‐term implications of a low dose of fluoxetine on the adult skeleton and body composition.

## AUTHOR CONTRIBUTIONS

Hannah P. Fricke, Julia F. Charles, and Laura L. Hernandez conceived of the experiments, analyzed, and interpreted the results. Lella A. Wake and Julia F. Charles analyzed bone microCT data. Katelyn M. Desorcy‐Scherer conceived and interpreted results. Hannah P. Fricke, Chandler J. Krajco and Molly J. Perry collected data. All authors read and approved the manuscript.

## FUNDING INFORMATION

This work was funded by NICHD R01HD094759 to Laura L. Hernandez. Hannah P. Fricke was funded by a T32HD041921. Julia F. Charles is funded by NIAMS R01AG046257 and R21AR07768.

## CONFLICT OF INTEREST STATEMENT

The authors have nothing to disclose.

## DISCLAIMERS

The authors have nothing to disclaim.

## Data Availability

All data are presented in the manuscript.
